# CaMKII Isoforms in Learning and Memory: Localization and Function

**DOI:** 10.3389/fnmol.2018.00445

**Published:** 2018-12-04

**Authors:** Gisela Zalcman, Noel Federman, Arturo Romano

**Affiliations:** ^1^Instituto de Fisiología, Biología Molecular y Neurociencias, Universidad de Buenos Aires – Consejo Nacional de Investigaciones Científicas y Técnicas, Buenos Aires, Argentina; ^2^Departamento de Fisiología, Biología Molecular y Celular, Facultad de Ciencias Exactas y Naturales, Universidad de Buenos Aires, Buenos Aires, Argentina

**Keywords:** CaMKII, learning, memory, CaMKIIα, CaMKIIβ, CaMKIIδ, CaMKIIγ

## Abstract

Calcium/calmodulin-dependent protein kinase II (CaMKII) is a key protein kinase in neural plasticity and memory, as have been shown in several studies since the first evidence in long-term potentiation (LTP) 30 years ago. However, most of the studies were focused mainly in one of the four isoforms of this protein kinase, the CaMKIIα. Here we review the characteristics and the role of each of the four isoforms in learning, memory and neural plasticity, considering the well known local role of α and β isoforms in dendritic terminals as well as recent findings about the γ isoform as calcium signals transducers from synapse to nucleus and δ isoform as a kinase required for a more persistent memory trace.

## Introduction

CaMKII is one of the main effectors enzymes involved in calcium signaling in eukaryotic cells. The enzyme is activated as a result of increased intracellular calcium and phosphorylates target proteins involved in various processes such as mobilization of synaptic vesicles, modulation of ion channels, regulation of gene expression, regulation of muscle contraction, and LTP (Wu and McMurray, 2001; Lisman et al., 2012; Ojuka et al., 2012). Over the last two decades CaMKII has become one of the most studied proteins in the nervous system, and it has proven to be a key protein involved in learning, memory, and synaptic plasticity (Lisman et al., 2002, 2012; Irvine et al., 2006; Lucchesi et al., 2011; Coultrap and Bayer, 2012). CaMKII is a holoenzyme composed of 12 subunits of 56–60 kDa that are assembled into 2 rings of 6 subunits each (Gaertner et al., 2004). Interestingly, these subunits are proteins encoded by four distinct but highly related genes termed *camk2a*, *camk2b*, *camk2d*, and *camk2g*, which give rise to four different CaMKII isoforms: α, β, δ, and γ. Each isoform has different calcium trapping kinetics, sub-cellular localization and affinity for other protein binding, thus enabling CaMKII to have different properties according to its subunit composition, which can be composed of a single type or a combination of isoforms (Srinivasan et al., 1994; Brocke et al., 1999). Furthermore, the RNAs that code for the different isoforms can undergo alternative splicing, resulting in the synthesis of approximately 30 different variants (Hudmon and Schulman, 2002). In the present review, we will first describe general features of CaMKII structure and mechanism of activation and then summarize the main features of each isoform and their splicing variants as well as our current understanding on their role in learning and memory. Studies focused on the interplay between subunit composition and functional outcome will not only contribute to understand why CaMKII is so fundamental for learning and memory processes but they might also reveal key information on the molecular mechanisms involved in memory storage.

## CAMKII Structure and Mechanism of Activation

**FIGURE 1:**
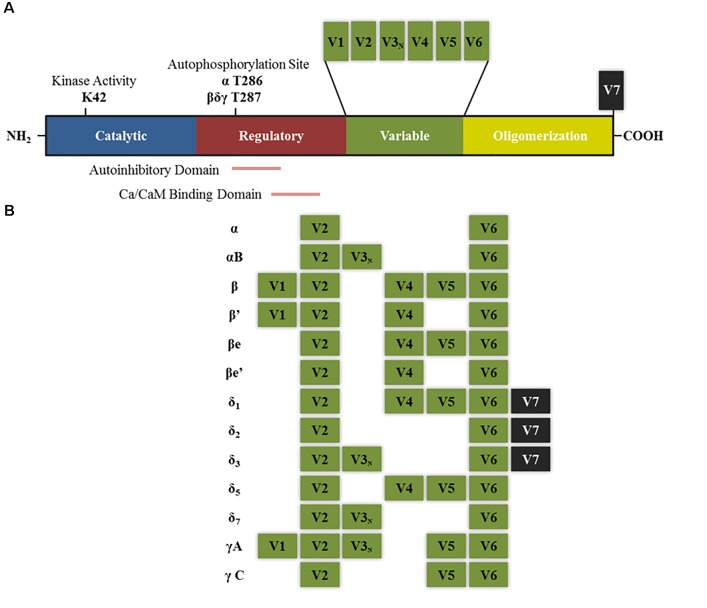
Structure of CaMKII isoforms and splice variants. **(A)** General structure of CaMKII protein including the different functional domains and main key regulatory sites. K42 denotes the lysine residue that allows ATP binding. T286 and T287 are the threonine residues that are autophosphorylated upon calcium binding. Other sites not mentioned in the text are T305–T306 present in the Ca/CaM binding domain, which can be phosphorylated after CaMKII autophosphorylation and negatively regulates its activity by preventing further calcium binding (Coultrap and Bayer, 2012). **(B)** Splice variants for α, β, δ, and γ isoforms that have been described in neurons. V denotes variable region and V3_N_ represents the variable region containing the nuclear localization signal. “e” Denotes that these isoforms are expressed in embryonic stages (adapted from Tombes et al., 2003).

Each of the subunits that comprise CaMKII multimeric enzyme has a conserved structure among the different isoforms: an amino terminal catalytic domain, followed by a regulatory domain that contains a self-inhibitory region and a binding site for the Ca^2+^/calmodulin (CaM) complex, a variable sequence and finally an associative (or oligomerization) domain in the carboxy-terminal end which allows assembly between the different subunits (Figure 1A; Schulman et al., 1995; Coultrap and Bayer, 2012). The homology in the catalytic and regulatory domains between the different isoforms and variants of splicing is 89–93%, the main differences between sequences are found within the variable domain (Hudmon and Schulman, 2002). The enzyme is expressed mainly in the brain, but also in the rest of the tissues. In some regions of the brain, such as the hippocampus, the protein levels reach up to 2% of total proteins (Erondu and Kennedy, 1985; Hudmon and Schulman, 2002).

The entry of calcium into the cell leads to the formation of the Ca^2+^/CaM complex, which binds approximately 3–4 calcium ions per CaM in a cooperative form (Hanson et al., 1994). This complex binds to the regulatory region of CaMKII and produces a conformational change, which not only leads to the phosphorylation of its substrates, but also to an inter-subunit, intra-holoenzyme autophosphorylation at threonine 286 in the α isoform and threonine 287 in β, δ, and γ isoforms. Autophosphorylation on this site prevents the enzyme to revert to its inactive conformation and decreases the dissociation rate of the bound CaM. Thus, autophosphorylated CaMKII can remain active even after the intracellular calcium levels decrease and therefore acquire autonomous and Ca^2+^ independent activity (Hanson et al., 1989, 1994; Hudmon and Schulman, 2002). Interestingly, this autonomous activity can persist even upon degradation or dephosphorylation of some subunits given they can be replaced and rephosphorylated by neighboring autophosphorylated subunits (Lisman et al., 2002; Irvine et al., 2006). The discovery of CaMKII mechanism of activation resulted in an increasing interest from the learning and memory field in this enzyme. First, because of the ability to detect small variations in the concentration of intracellular calcium, as action potentials in neurons involve repeated pulses of calcium and memory formation is also tightly linked to the activation of calcium channels like NMDA and Cav1 (Morris, 2013; Berger and Bartsch, 2014). Second, because its singular ability to acquire persistent autonomous activity has been proposed as a form of molecular memory (Lisman et al., 2002). Indeed, subsequent studies have proven that CaMKII has a key role in memory as we will describe below.

## CAMKII and Memory

The activation of the NMDA receptor in glutamatergic excitatory pathways following a behavioral experience is key for long term memory (LTM) storage (Morris, 2013). This activation allows the entry of calcium into the neuron which binds to calmodulin to form the Ca^2+^/CaM complex that is recognized by multiple enzymes, thus inducing a molecular signaling cascade whose main function is to reshape the synaptic structure and physiology, together with regulation of gene expression necessary for the formation of LTM (Giese and Mizuno, 2013). CaMKII is one of the main targets of Ca^2+^/CaM. Accordingly, CaMKII activity is increased upon learning and its inhibition causes LTM impairment (Tan and Liang, 1996; Lucchesi et al., 2011; for reviews see Cammarota et al., 2002). Learning to an inhibitory avoidance task increased hippocampal CaMKII activity up to 30 min after training (Cammarota et al., 1998). Studies in synaptic plasticity have shown that CaMKII activity after LTP induction seems to be increased only 1–2 min (Lee et al., 2009; Chang et al., 2017). This kinetics in CaMKII activity does not match that observed memory, suggesting that LTP may be regulated differently than memory formation. For instance, the longer kinetics observed in behaving animals experiments could suggest that memory formation may involve several “rounds” of LTP-like processes, and not just a single one.

To further understand the role of CaMKII in learning and memory processes different transgenic mice lines have been generated. Yasuda and Mayford (2006) developed a transgenic mouse line in which transgene expression of a constitutively active mutant form of CaMKII was inducible and limited to the superficial layers of medial entorhinal cortex, pre- and parasubiculum (Yasuda and Mayford, 2006). The transgene was constitutively expressed and could be inhibited after the administration of doxycycline. Learning to find a visible platform in a water maze was similar across trials between wild-type and transgenic mice, however, long-term memory formation of the platform location, as assessed in a testing session 24 h after training, was impaired in transgenic animals. Mice were also trained to novel object recognition task, and transgenic mice showed recognition memory impairment when tested 3 h after training, thus short-term memory storage for this non-spatial task was also impaired. Finally, using doxycycline to inhibit transgene expression, they performed two different experiments: (i) transgene was expressed immediately after testing to the water maze task and up to 3 weeks after, (ii) transgene was expressed 3–6 weeks after training. These allowed studying the role of CaMKII activity after long-term memory consolidation. In the first case memory was impaired when tested 6 weeks after, suggesting that manipulating CaMKII activity affected the maintenance of the memory trace. In the latter, memory was not affected when tested 6 weeks after, indicating that at longer retention periods memory may have undergone systems consolidation and could be allocated in other cortical structures. More recently, a genetically encoded light-inducible inhibitor of CaMKII, called paAIP2, was designed, which allowed a tight temporal control on the manipulation of CaMKII activity. Mice received an intra-amygdala injection of a virus containing the vector for the expression of paAIP2 and were implanted an optic fiber guided to the amygdala. Inhibiting CaMKII activity during double-trial training to an inhibitory avoidance task led to memory impairment when tested 1 h after. Its inhibition immediately after training had no effect on memory when tested 1 h after training. From these results the authors concluded that CaMKII activity in the amygdala during training, but not after, is necessary for memory formation. It should be noted that, since the testing session took place 1 h after training, they evaluated short-term and not long-term memory storage (Murakoshi et al., 2017).

CaMKII binds to numerous proteins in the post-synaptic density (PSD), including NMDA receptor, synapsin 1, F-actin, and calcium channels. Presumably, the binding and phosphorylation regulates autonomous activity, location and/or transport of these and other proteins to regions of interest (Lucchesi et al., 2011; Sanhueza and Lisman, 2013; Hell, 2014). CaMKII can also regulate the number of AMPA receptors, together with their conductance, promoting the rapid growth of dendritic filopodia and spine formation (Jourdain et al., 2003; Lucchesi et al., 2011; Giese and Mizuno, 2013).

An important issue regarding neural plasticity and memory is the role of CaMKII in synaptic tagging and capture (STC) and behavioral tagging. STC is one of the proposed mechanisms that explain how the plasticity-related proteins (PRP) act only in the activated synapses by means of an activity dependent tag that allows the capture of PRPs in these specific synapses. CaMKII have been proposed as a tag mechanism for LTP (Sajikumar et al., 2007) and for long-term depression (LTD) (Szabó et al., 2016). In relation with the first finding, Redondo et al. (2010) found that the tag in LTP is sensitive to CaMKII inhibition. Such tagging function of CaMKII is transient and is then replaced by a PKMζ-mediated mechanism dependent on ryanodine receptor or synaptic activation of metabotropic glutamate receptors that prolongs the durability of the synaptic tag (Li et al., 2012). In studies with behaving animal the learning tag is setting by activation of the glutamatergic NMDA receptors and this machinery further required CaMKII and PKA but not ERK1/2 protein kinase activity (Moncada et al., 2011).

## CAMKII Isoforms

δ and γ are isoforms are expressed in different tissues including the brain while α and β isoforms are brain specific (Bennett et al., 1983; Tobimatsu and Fujisawa, 1989). The studies on each isoform allow distinguishing differences and similarities in their affinity for calcium, their location and function. For instance, affinity for calmodulin and the rate of autophosphorylation between the different isoforms is estimated to be γ > β ∼ δ > α and δ > β > α > γ, respectively (Gaertner et al., 2004). Moreover, all the isoforms contain different splicing variants which have different sub-cellular localization and protein binding affinity, and thus even if the holoenzyme is only composed of subunits from a same isoform (homomeric), its overall features and function will depend on the properties of the variants that contains (Srinivasan et al., 1994).

## CaMkiiα

**FIGURE 2:**
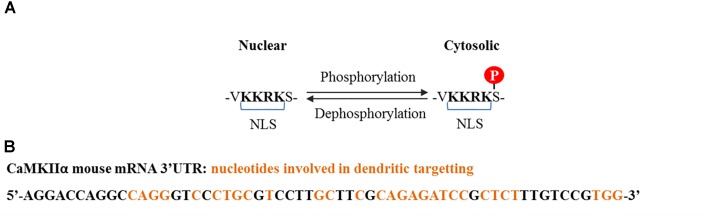
CaMKIIα nuclear localization signal and dendritic mRNA location sequences. **(A)** Nuclear localization signal contained by some splice variants from α, δ, and γ isoforms. Nuclear translocation is regulated by phosphorylation at the serine adjacent to the NLS. Phosphorylation at this serine by CaMKI and CaMKIV prevents CaMKIIα and CaMKIIδ nuclear translocation (Heist et al., 1998), whereas dephosphorylation, presumably by PP2A for CaMKIIδ and PP2B (calcineurin) for CaMKIIγ, promotes translocation (Zhang et al., 2002; Ma et al., 2014). **(B)** Common cis elements that have been shown to be involved in CaMKIIα dendritic mRNA location and which are conserved among rat, mouse, and human (Mori et al., 2000).

CaMKIIα contains three variants termed α, αB, and αKAP, the first two are abundant in the brain while the last one is expressed in skeletal muscle (Hudmon and Schulman, 2002). The αβ variant contains a nuclear localization signal (NLS) which is also shared with variants of the δ and γ isoforms, and is liable to regulation (Figure 2A). The mRNA for α variant is expressed throughout the brain, while α_B_ mRNA is restricted to diencephalon/midbrain regions. Immunohistochemical studies performed in rat brain revealed that indeed, in thalamic and hypothalamic neurons which express both variants, CaMKIIα protein localizes to the nucleus and the cytoplasm, while in caudate putamen, in which only the α variant is expressed, CaMKIIα was exclusively seen in the cytoplasm (Brocke et al., 1995). The αKAP variant lacks the catalytic domain but contains a domain that facilitates its binding to the sarcoplasmic reticulum and a NLS, although its sub-cellular localization is mainly ruled by the membrane targeting sequence (Hudmon and Schulman, 2002), serving as an anchoring protein of catalytic competent subunits to membranes.

In neurons, CaMKIIα localizes in pre-synapses and dendrites and is actually one of the main proteins in the PSD (Griffith et al., 2003; Liu and Murray, 2012). Its location in PSD is calcium-dependent and it has been shown to be induced after NMDA (but not AMPA or metabotropic glutamatergic) receptor stimulation and is regulated by CaMKIIβ F-actin binding (Shen and Meyer, 1999; Ahmed et al., 2006, see also CaMKIIβ section). Also it is almost exclusive of synapses receiving glutamatergic terminals (Liu and Jones, 1996, 1997; Liu and Murray, 2012). In cultured hippocampal neurons expressing a mutant form of CaMKIIα with impaired kinase activity or T286 autophosphorylation, PSD translocation after glutamate stimulation was not altered compared to CaMKIIα wild type, and a CaMKII mutant that simulates the autophosphorylated state did not localize to PSD before stimulation, suggesting that neither kinase activity nor autophosphorylation are necessary for CaMKIIα translocation to PSD. However, it was shown that after external calcium and glutamate removal the mutant CaMKIIα with impaired autophosphorylation dissociated more rapidly from the PSD than CaMKIIα wild type (Shen and Meyer, 1999). Thus neither kinase activity nor T286 autophosphorylation are necessary for PSD translocation, but the later can regulate the process.

Of particular interest is a 30 nucleotide sequence present in the 3′ UTR of the CaMKII mRNA that targets it to dendrites, where it is locally translated (Figure 2B; Mayford et al., 1996). Its transport is inhibited in a resting state and is activated upon neuronal depolarization and NMDA receptor stimulation (Mori et al., 2000; Blichenberg et al., 2001; Néant-Fery et al., 2012). These particular characteristics and the fact that the PSD is a key compartment for the molecular events that subserve memory storage turned CaMKIIα to be the most studied isoform in the field of neurobiology of memory.

## Role of α Isoform in Memory

Different CaMKIIα mutant mice have been generated, including homozygous and heterozygous knock out (KO) mice (Silva et al., 1992a,b, 1996), KI mice with impaired Ca^2+^/CaM binding (Elgersma et al., 2002), KI mice with a mutation blocking the catalytic activity of CaMKIIα (Yamagata et al., 2009), KI mice with a mutation in T286 impeding autophosphorylation (T286A) (Giese et al., 1998) and transgenic mice expressing a mutated form of CaMKIIα that can be rapidly and reversibly inhibited after the administration of a synthetic inhibitor (Wang et al., 2003). The effects of these and other mutations on memory and synaptic plasticity has been reviewed in Elgersma et al. (2004), Irvine et al. (2006), and Giese and Mizuno (2013), here we will present the main findings.

Homo and heterozygous KO mice showed learning impairment in several hippocampal-dependent tasks which could be sometimes overcome with extended training (Silva et al., 1992a; Yamasaki et al., 2008). However, these transgenic animals had the side effect of showing abnormal behavior, including altered locomotors activity and anxiety-like behavior, as well as an increased targeting of Cambia to PSD which could be compensating for the loss of CaMKIIα (Elgersma et al., 2004; Yamasaki et al., 2008). Subsequent studies were done in transgenic mice bearing knock-in mutations on different key regions of the endogenous CaMKIIα gene. KI transgenic mice that express a form of CaMKIIα lacking its kinase activity showed learning and consequent memory impairment in a one-trial inhibitory avoidance task, while KI animals with impaired autophosphorylation at T286 had learning and memory impairments in one-trial passive avoidance tasks, the water Morris maze and contextual fear conditioning (Giese et al., 1998; Irvine et al., 2005; Yamagata et al., 2009). Learning impairment could be overcome with repeated training and, in the case of animals with impaired autophosphorylation at T286, this also rescued memory deficits (it should also be noted that in these animals CaMKII Ca^2+^-independent activity was reduced by 60% but not completely, so there could be some compensatory effect from the activation of other isoforms). Thus, it was proposed that autophosphorylation at T286 could be related to one-trial learning and not necessarily to memory storage (Giese and Mizuno, 2013). Regarding the need of CaMKIIα activity in memory storage, it has been shown to be necessary for inhibitory avoidance LTM formation even after repeated training. On the other hand, heterozygous KO mice showed normal contextual fear conditioning learning and LTM 1–3 days after training but this was severely impaired at longer retention delays, when memory becomes independent of hippocampal processing and becomes dependant on storage in the cortex. Therefore, these results suggest a role of cortical CaMKIIα in systems consolidation (Frankland et al., 2001, 2004).

In non-transgenic animals it has been shown that CaMKIIα expression is also regulated in different types of learning and brain structures. For instance, in the hippocampus, a gene screening (coda array) showed that CaMKIIα mRNA was up-regulated 3 h but not 24 h after one-trial inhibitory avoidance training and its protein levels were increased 24 h but not 3 h after training (Igaz et al., 2004). In the dentate gyrus, western blot analysis revealed that object-place recognition learning triggered an increment in CaMKIIα protein expression 1 h after training. In the striatum, permanent inhibition of CaMKIIα expression after the stereotaxical injection of a lentivirus expressing a shRNA before training impaired accelerating rotarod and water cross maze test performances. However, these animals were able to learn properly after consecutive training similarly to what it has been seen for transgenic animals (Wang et al., 2017).

A key feature of CaMKIIα relies on its local translation in dendrites. Regarding the role of this process in memory, it has been shown that when the mRNA was confined to the soma by mutation of the 3′-untranslated region in mice, LTM, but not short term memory nor learning, was impaired in cued and contextual fear tasks, water Morris maze and an olfactory associative task (Miller et al., 2002; Néant-Fery et al., 2012). These results point to an important role of CaMKIIα local translation in LTM formation.

Altogether, these findings support that CaMKIIα proper catalytic activity and T286 autophosphorylation are important for enabling fast learning, that its gene expression is involved in memory consolidation and that this protein may also have a role in remote memory formation.

## CaMkiiβ

CaMKIIβ isoform possesses six splicing variants (Figure 1). The ones that have been described in the brain are β, β′, βe and β′e (Brocke et al., 1995; Hudmon and Schulman, 2002; O’Leary et al., 2006; Cook et al., 2018). A recent publication has also described the existence of a new variant expressed in the brain which was termed βH (Cook et al., 2018). None of the splice variants contain a NLS and this could explain why this isoform has not been shown to be present in the nuclear compartment.

**FIGURE 3:**
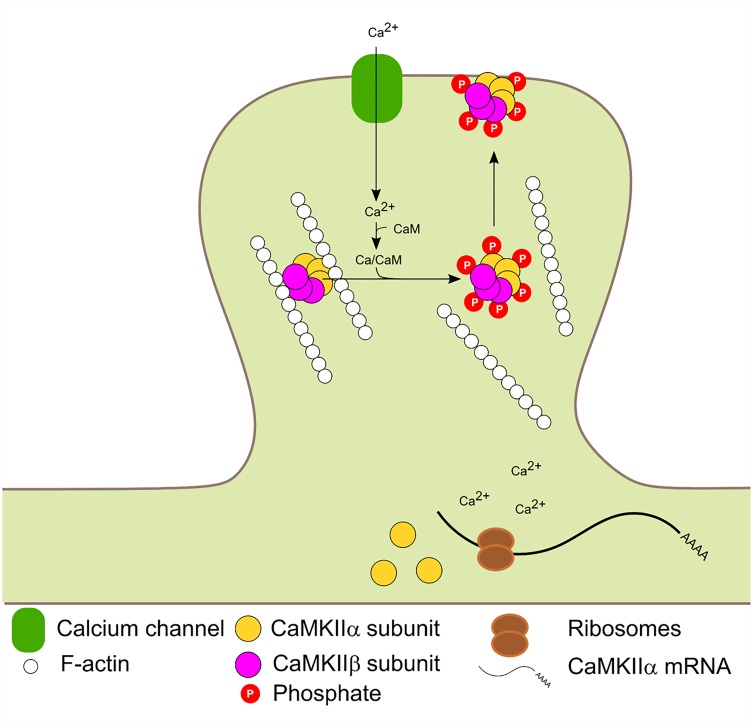
Activity dependent PSD translocation and CaMKIIα dendritic translation. Synaptic activity leads to Ca^2+^ influx. Subsequent Ca^2+^/CaM binding to CaMKIIα and β induce F-actin dissociation, translocation to the PSD and autophosphorylation at T286 and T287 in α and β isoforms, respectively. Calcium entry also enhances local CaMKIIα mRNA translation.

The β and β′ variants contain an F-actin binding domain and are therefore, able to bind F-actin in basal conditions. Glutamate postsynaptic stimulation induces CaMKIIβ/F-actin release and subsequent PSD localization. The dissociation process requires binding of Ca^2+^/CaM to CaMKIIβ and/or autophosphorylation at T287 and is not dependant on CaMKIIβ kinase activity (Shen and Meyer, 1999; Lin and Redmond, 2008). Mutated forms of CaMKIIβ with impaired calmodulin binding could bind to F-actin in basal conditions but were unable to dissociate after stimulation. A mutated CaMKIIβ which mimic the autophosphorylation state could not bind to F-actin. Thus F-actin binding requires the enzyme to be in a non-autophosphorylated state (not active) and its dissociation requires Ca^2+^/CaM binding. CaMKIIβ is usually associated with CaMKIIα to form the heterozyme α/β-CaMKII which translocates jointly to the PSD. Interestingly, translocation kinetics is dependent on the ratio of α/β subunits in the heteromer (Shen and Meyer, 1999). It has also been proposed that the δ and γ isoforms can bind F-actin *in vitro* (Hoffman et al., 2013). Binding of CaMKIIβ to F-actin has been shown to increase F-actin stabilization and bundling (O’Leary et al., 2006; Okamoto et al., 2007; Lin and Redmond, 2008; Sanabria et al., 2009) and was proposed that CaMKIIβ transient dissociation from F-actin promotes actin re-organization which impacts in dendritic spine remodeling (Okamoto et al., 2007; Giese and Mizuno, 2013). Figure 3 summarizes CaMKIIβ and α molecular dynamics in the PSD.

CaMKIIβ has been implicated in a process of inverse synaptic tagging, targeting of Arc to inactive synapses via a high-affinity interaction with the β isoform that is not bound to Ca^2+^/CaM (Okuno et al., 2012).

## Role of β Isoform in Memory

A transgenic mice line was developed which expressed a mutated form of CaMKIIβ whose activity could be reversibly inhibited upon the administration of a synthetic inhibitor (Cho et al., 2007). This over-expression of CaMKIIβ led to an increase in its enzymatic basal activity. In the hippocampus, CaMKIIβ over-expression was seen exclusively in the dentate gyrus. Learning and 1 day retention for novel object recognition task, cued and contextual fear conditioning were normal, but in this last task, 10 days retention was impaired (Cho et al., 2007). These animals also presented reversal learning deficits in the radial arm maze task and the water cross maze (Yin et al., 2017). Upon administration of the synthetic inhibitor at different time points before and after training, the authors could further conclude that the 10-day memory impairment was a consequence of increased CaMKIIβ expression during the LTM consolidation phase, and not during acquisition or recall. The fact that the memory deficit was observed 10 days but not 24 h after training indicates that CaMKIIβ activity during consolidation modulates the persistence of the memory that is being consolidated. On the other hand, two KO mice for CaMKIIβ were developed, which differed in the locus in which a neomycin cassette was inserted to disrupt CaMKIIβ gene sequence, and where later used to study the role of CaMKIIβ in memory (van Woerden et al., 2009; Bachstetter et al., 2014). In one of these studies, animals showed impairment in novel object recognition when tested 4 h after training, suggesting CaMKIIβ could be involved in short-term memory formation. These animals also showed differences compared to wild type in rotarod tests, balance beam and running wheel tasks, elevated plus maze and open field and they had altered body mass composition, among others traits (Bachstetter et al., 2014). In another of this studies KO mice showed impaired fear conditioning memory when tested 24 h after training (Borgesius et al., 2011). Interestingly, in this last work the authors found that CaMKIIα expression levels were not altered in the CaMKIIβ KO mice but its location to synapses was decreased by a 40%. This prompted the authors to study if the memory deficits were a side-effect of decreased CaMKIIα localization in PSD. To test this they used a KI mouse line in which Ca^2+^-dependent activation is blocked by interfering with Ca^2+^ binding, thus in these animals CaMKIIβ can bind to F-actin but cannot be activated. Fear conditioning performance was normal in these animals and CaMKIIα abnormal distribution was not seen. This last result led to the important conclusion that CaMKIIβ binding to F-actin but not its catalytic activity is necessary for memory processes and CaMKIIα translocation to spines.

Little is known regarding CaMKIIβ expression changes in memory processes. In the striatum, it has been shown that CaMKIIβ mRNA expression was up-regulated specifically after extensive training to an accelerated rotarod task, suggesting a delayed effect of training on CaMKIIβ expression (D’Amours et al., 2011).

Altogether, these results suggest that β isoform has a non-enzymatic role in LTM consolidation. Presumably a key function of this enzyme is to allow CaMKIIα translocation to the PSD. However, the activity-dependent dissociation to F-actin seems to be important to regulate actin dynamics that may allow dendritic spine plasticity and synaptic reorganization (Okamoto et al., 2007; Hotulainen and Hoogenraad, 2010; Lamprecht, 2014). This fact could explain why manipulating CaMKIIβ activity during the consolidation phase can affect memory persistence several days after, as seen in Cho et al. (2007).

## CaMkiiδ

The δ isoform is expressed ubiquitously. To date, 13 different variants have been identified from alternative splicing of the gene which are expressed differentially in the brain, heart, and skeletal muscle. The structures of 10 of these variants have been studied in detail and show that the main difference between them is within two variable regions located next to the regulatory domain and the C-terminal end of the association domain, respectively (Mayer et al., 1993, 1995; Zhang and Brown, 2004; Figure 1B). The variants 1, 2, 3, 5, and 7 have been shown to be expressed in the brain (Schworer et al., 1993; Kamata et al., 2006). Interestingly, the variants 3 and 7 contain a NLS which is conserved with the isoforms α and γ (Srinivasan et al., 1994). The expression of 2, 3, and 7 variants are highly abundant in heart tissue, where they have been shown to be involved in ischemia/reperfusion injury and the activation of NF-κB signaling cascade (Gray and Heller Brown, 2014; Gray et al., 2017). A recent publication has shown that this kind of injury in the brain also triggers a similar signaling cascade (Zhang et al., 2012). In brain, CaMKIIδ protein has been shown to be present in the nucleus of cerebellar granule cells and substantia nigra dopaminergic neurons in rats (Takeuchi et al., 1999; Kamata et al., 2006). It has been proposed that CaMKIIδ nuclear translocation in neurons is promoted by PP1-dependent dephosphorylation at Ser 332 enhancing BDNF transcription (Kamata et al., 2006; Shioda et al., 2015). More recently, we found the δ isoform in mice hippocampal pyramidal cells, with an important presence in nuclei (Zalcman et al., 2018).

We recently found in the hippocampus that CaMKIIδ is also located in pre-synaptic terminals. Even though strong evidence points to a crucial role for hippocampal pre-synaptic CaMKII in synaptic plasticity and neurotransmitter release (Jovanovic et al., 2001; Ninan and Arancio, 2004; Lu and Hawkins, 2006; Wang, 2008), little is known about the presence of each isoform in this compartment. The presence of CaMKIIδ in axons has been documented during neuronal development. Effectively, CaMKIIδ is the main isoform present in the axons of developing rodent neurons, related with neurite outgrowth and stability (Donai et al., 2000; Johnson et al., 2000; Faison et al., 2002; Easley et al., 2006).

## Role of δ Isoform in Memory

Until recently, the role of CaMKIIδ in memory processes was virtually unknown. In a first study from our laboratory, we showed that training to a Novel Object Recognition (NOR) task induced NF-κB binding and histone acetylation in the promoter of δ isoform gene as well as an increase in its expression 3 h after training (Federman et al., 2013). NF-κB is a key transcription factor for long-term memory consolidation (Alberini, 2009; Romano, 2012; de la Fuente et al., 2015). Histone acetylation is a molecular process that enhances gene expression and is also related to memory strength and persistence (Federman et al., 2013, 2014; Kim and Kaang, 2017). This first evidence led us to the hypothesis that CaMKIIδ gene expression could be sustained beyond memory consolidation in order to warrant memory persistence. More recently, we found that, indeed, training to a NOR task increased CaMKIIδ mRNA levels several days after training (Zalcman et al., 2018). Its expression was increased up to 7 days while memory retention was present, and returned to basal levels 20 days after, when recognition memory retention was vanished. These results suggest that CaMKIIδ expression parallels memory retention. This persistent gene expression was also accompanied by long-term changes in nucleosome dynamics on key sites of its promoter, further supporting the presence of long-term molecular events to regulate its expression. Such a sustained gene expression after learning has been rarely reported, with the exception of PKMζ, a protein that has been shown to be up-regulated up to 7 days after training in the insular cortex (Shema et al., 2011) and for 1 month in the hippocampus (Hsieh et al., 2016). Therefore, has been proposed to participate in memory maintenance. In accordance with these findings, inhibiting CaMKIIδ gene expression 24 h after NOR training, once memory has been consolidated, led to memory impairment at day 7, suggesting that CaMKIIδ is indeed involved in mechanisms that affect maintenance of the memory trace. The fact that inhibition of CaMKIIδ gene expression during consolidation affect memory at 7 days but left 1 day memory intact support a specific action in persistence and maintenance. Previous evidence has implicated CaMKII activity in memory maintenance. In a recent publication it was shown that transient hippocampal expression of an inactive form of CaMKII 3 days after training, once memory is supposed to have been consolidated, affected behavioral performance when tested 9 days after training, a time point in which virally mediated protein expression had ended (Rossetti et al., 2017). On the other hand, it was also shown that the expression of a constitutively active mutant form of CaMKII in parahippocampal regions, during the first 3 weeks after training to a water maze task, affected the maintenance of a previously established spatial memory (Yasuda and Mayford, 2006). On the contrary, inhibiting CaMKII activity 24 h after training to an inhibitory avoidance task, had no effect on a behavioral test performed 1 h after the inhibition (Buard et al., 2010). This last result, taking into account the previous two, suggest that while CaMKII activity may be necessary for memory maintenance, it may not have an immediate impact on the memory trace, thus the impairment may be observable several days after the manipulation.

**FIGURE 4:**
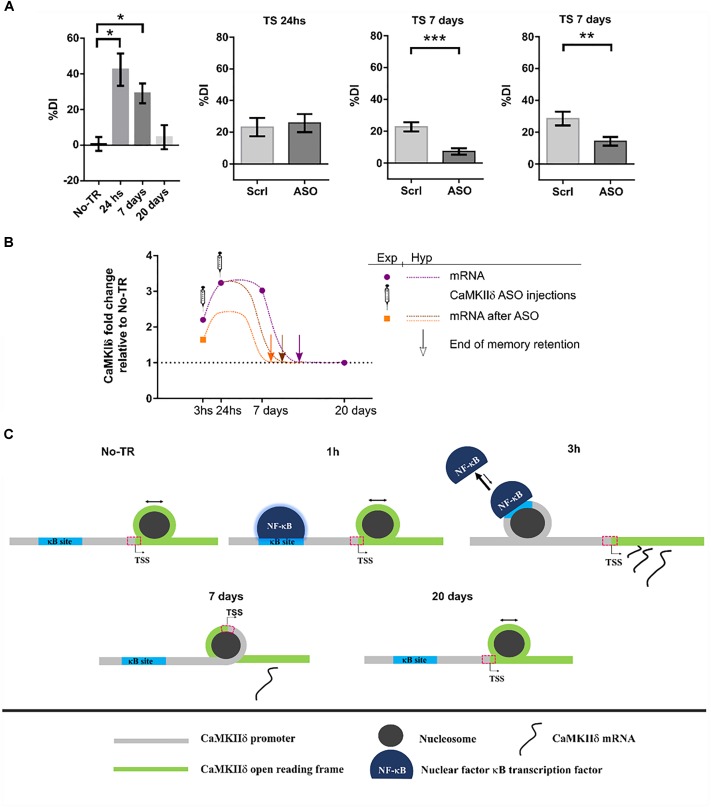
CaMKIIδ role in memory persistence. **(A)** Left graph shows mice performance 24 h, 7 days, and 20 days after training to Novel Object Recognition task compared to non-trained animals (No-TR). Performance is expressed as a discrimination index (%DI) that is an indicator of the time spent exploring a novel object over a familiar one (Zalcman et al., 2018). Note that memory retention decays with time and is significantly above the control group when animals are tested 24 h and 7 days after training. Infusion of an oligonucleotide antisense to CaMKIIδ mRNA (ASO), which effectively decreases CaMKIIδ expression, 2 h after training leads to memory impairment when animals were tested 7 days after but not 24 h (middle graphs). Seven days memory impairment was also found when CaMKIIδ ASO was injected 24 h after training (right graph). Scrl: scrambled oligonucleotide used as a control (with permission of Springer Nature). **(B)** Graph showing the mRNA levels for CaMKIIδ measured experimentally at different time points after training and after ASO administration (purple circles and orange square, respectively) together with a hypothetical curve (dash lines). Based on our results, we propose that memory can be recalled as long as CaMKIIδ mRNA levels are above basal condition. Arrows indicate a hypothetical time point at which CaMKIIδ mRNA return to basal levels and thus memory retention is lost. ASO administration shifts mRNA curve in such a way that only 7 days memory is affected. **(C)** Nucleosome occupancy on κB and transcription start sites (TSS) is affected by training to a NOR task. One hour after training NF-κB is bound to its κB site in CaMKIIδ promoter but there is no change in nucleosome occupancy at this site compared to non-trained animals (Federman et al., 2013; Zalcman et al., 2018). Three hours after training nucleosome occupancy is increased in κB site, and it is decreased on TSS site, there is also an increase in CaMKIIδ transcription. Since nucleosome occupancy at specific regions has been proposed to disfavor protein binding (Bai and Morozov, 2010), the observation at 3 h suggests that NF-κB binding decreases while CaMKIIδ transcription is enhanced. Seven days after training nucleosome occupancy at TSS is increased presumably decreasing the rate of gene expression. Finally, 20 days after, when memory retention is lost, nucleosome occupancy on TSS site becomes return to the level of non-trained animals. ^∗^*p* < 0.05; ^∗∗^*p* < 0.005; ^∗∗∗^*p* < 0.0005.

Interestingly, whilst it is not common to observe sustained gene expression for most proteins, this seems to be a feature of CaMKIIδ. It has been shown that CaMKIIδ protein expression can be increased up to 5 days after brain injury in rats (Zhang et al., 2012) and up to 7 days in homogenates form ventricles after transverse aortic constriction (Zhang et al., 2003). CaMKIIδ mRNA was also up-regulated 7 and even 21 days after nerve injury in the peripheral system (Xiao et al., 2002; Bangaru et al., 2015). Moreover, what it is remarkable from our results is not only the novelty on CaMKIIδ involvement in memory persistence, but also that it poses two novel molecular mechanisms by which a memory trace is allowed to persist on time: sustained gene expression and nucleosome positioning dynamics. Figure 4 summarizes our findings and proposes a general mechanism by which a stronger training favors the formation of a more persistent memory trace.

At this point two key questions arise. First, in which way is CaMKIIδ gene expression functional to memory maintenance? A maintenance molecular mechanism should be sustained over time and should be necessary for memory retention beyond the consolidation time window. In this report we provide evidence that support this role for CaMKIIδ. In addition, such mechanism should be involved in synaptic efficacy modifications. The action of CaMKIIδ in pre-synapses of excitatory pathways could determine an increment in neurotransmitter release (Wang, 2008) and its action in the nucleus could regulate epigenetic mechanisms and transcription of effectors genes. In cardiomyocytes nuclear translocation of CaMKIIδ has been shown to regulate histone H3 phosphorylation at serine 10. Besides, it induces histone deacetylase HDAC4 export to the cytoplasm, increasing gene transcription (Zhang et al., 2007; Awad et al., 2015). Notably, both H3 phosphorylation and HDAC4 have been related to memory processes, with HDAC4 being the most studied deacetylase subtype in neural plasticity and memory (Sando et al., 2012), and its role in this process evolutionarily conserved (Fitzsimons et al., 2013, p. 4; Reul, 2014; Hudson et al., 2015). Therefore, CaMKIIδ may be regulating these proteins in order to promote sustained changes in the expression of genes necessary to maintain the memory trace. The second question is how might CaMKIIδ sustain its gene expression? We propose that there is a feed-back mechanism in the nucleus, in which CaMKIIδ phosphorylates HDAC4 and histone H3 to sustain its own expression over time. Since CaMKIIδ mRNA expression returns to basal levels after 20 days, this feed-back mechanism may decay over time. This last assumption is in accordance with our finding that nucleosome occupancy at the *camk2d* transcription start site changed 7 days after training in an opposite manner to what was observed in an early phase (Zalcman et al., 2018).

## CaMkiiγ

**FIGURE 5:**
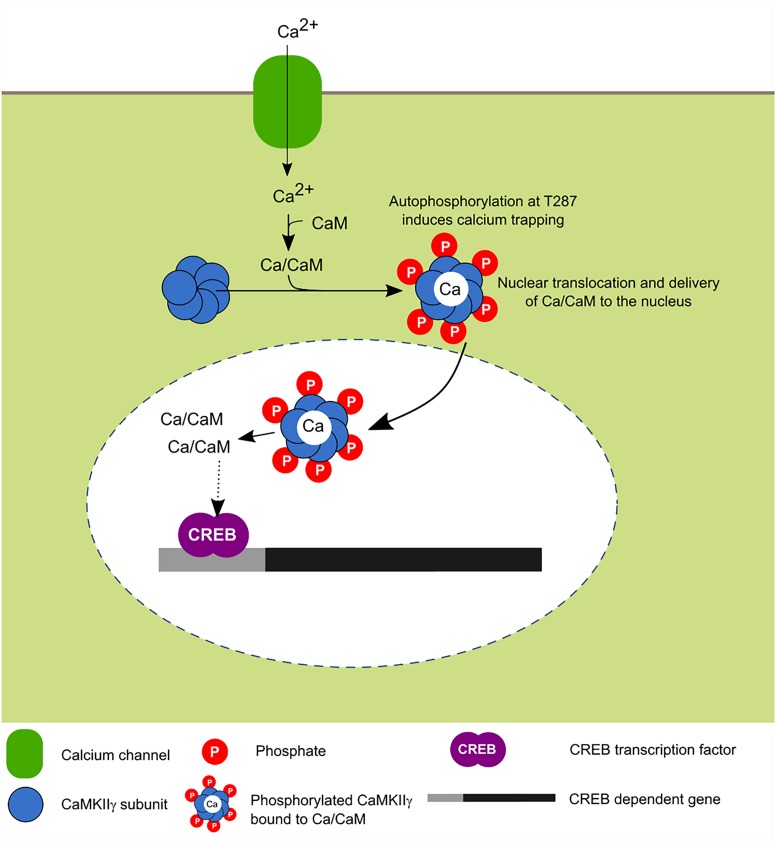
Activity-dependent nuclear translocation of CaMKIIγ. Recent findings propose that neuronal activity induces CaMKIIγ nuclear translocation whose main role is to shuttle Ca^2+^/CaM to the nucleus, this leads to the phosphorylation of CREB transcription factor which is associated with enhanced transcription (Ma et al., 2014; Malik et al., 2014).

The γ isoform is expressed in the brain of mammals, in cardiac tissue, in smooth muscle, liver, and cells of the immune system (Tobimatsu and Fujisawa, 1989; Bayer et al., 1999; Gangopadhyay et al., 2003). As with the other isoforms, CaMKIIγ can be spliced to different variants termed γA to γH (Hudmon and Schulman, 2002; Gangopadhyay et al., 2003). The γA variant is brain specific and contains the NLS, present in other CaMKII isoforms, thus can translocate to the nucleus by a mechanism similar to the one established for the other isoforms (Figure 5). Other variants, like γC, have also been shown to be expressed in neurons (Kamata et al., 2006).

In cultured neurons, it was recently shown that depolarization induces the translocation of this isoform to the nucleus and it has been postulated that its main function is to serve as a transport protein of the Ca^2+^/CaM complex to the nucleus in order to induce the signaling cascade dependent on this signal and activate CaM kinase families for the transcriptional regulation mediated in part by CREB (Ma et al., 2014).

## Role of γ Isoform in Memory

Few studies have been published showing a connection between this isoform and memory. In humans it has been shown that genetic variability around this gene affects performance in episodic memory tests (de Quervain and Papassotiropoulos, 2006) and intellectual capacity (de Ligt et al., 2012). CaMKIIγ KO mice have been recently developed. This animals showed learning deficits in Morris water maze and Radial arm maze, as well as impaired long-term memory in an inhibitory avoidance task (Cohen et al., 2018). These tasks are mainly hippocampal-dependent, while inhibitory avoidance is also dependent on the amygdala. It should be noted, that learning curves were analyzed over consecutive days, and is therefore difficult to disentangle a learning deficit from a long-term memory storage deficit. Normal anxiety and locomotor activity were not affected in mutant animals compared to wild type littermates. They further demonstrated that these animals had decreased levels of nuclear CaM and performed different experiments to show that this was an effect of deficient CaMKIIγ-dependent transport of CaM to the nucleus. Altogether these results show that CaMKIIγ is involved in spatial learning and long-term memory storage.

## Concluding Remarks and Future Directions

Although the key role of CaMKII in neural plasticity and memory have been stressed by decades of study since the initial discovery of its role in long-term potentiation (LTP), the specific role of each isoform in different subcellular compartment is still under investigation and required further analysis (Box 1). Recent findings suggest that each of the four isoforms play different roles and, in some cases, this role is not dependent on the enzymatic function. As was described earlier, the α isoform plays an important role associated with the PSD in dendritic spines of excitatory synapses, regulating receptor and channel functions as well as receptor trafficking. The β isoform could have a non-enzymatic role in LTM consolidation, presumably by allowing CaMKIIα translocation to the PSD. The activity-dependent dissociation to F-actin seems to be important to regulate actin dynamics that may allow dendritic spine plasticity and synaptic reorganization. The main function of γ isoform is attributed to a synapse-to-nucleus communication, transporting Ca^2+^ signaling to regulate gene expression that is essential for the neural plasticity involved in memory. Recent results support a key role of the δ isoform in memory persistence and maintenance by means of the sustained expression of its gene. The presence of CaMKIIδ in the presynapses and in the nucleus is an important finding which deserves further study.

**BOX 1. Open questions on CaMKII isoforms in learning and memory.**

**Which is the subunit composition of endogenous CaMKII in the brain and how does this affect its location and function?**

Little is known on the endogenous assembly of subunits and variants, how this affects the overall function and location of the enzyme and if this could be regulated by behavioral experience. Current evidence indicates that in forebrain, endogenous CaMKIIα forms homomers as well as heteromers with CaMKIIβ (Brocke et al., 1999), and that the percentage of each subunit on the holoenzyme affects autophosphorylation rate and PSD translocation (Shen et al., 1998; Brocke et al., 1999; Shen and Meyer, 1999). Further studies are necessary to learn about the formation of heteromers with the other isoforms, δ and γ, as well as splicing variants composition.

**What is the gene expression kinetics of the different isoforms during the different memory phases?**

Much of the work on the role of CaMKII expression in learning and memory has been done using transgenic animals. Gene expression is a key molecular event in the formation and storage of long-lasting memories, therefore further studies on the expression of the different isoforms will help to provide insight not only on their role in learning and memory but also on the molecular mechanisms that underlie these processes.

**What is the role of the different isoforms in pre-synaptic terminals and nucleus?**

Most of the studies in CaMKII have focused on synaptic plasticity at the post-synaptic terminal (Coultrap and Bayer, 2012; Hell, 2014). However, learning and memory also requires modifications at the presynapses and changes in gene transcription. CaMKII has been shown to be present in both compartments. Thus, further studies on the localization and function of each isoform in these compartments are required.

Beyond the individual role of each isoform, CaMKII is multimeric. The assembly of 6 and then 12 subunits into holoenzymes is an important structural feature that can regulate and modify the function and localization of the kinase. The mRNA of α isoform presents 3′ UTR sequences that target the messenger to dendrites, where it is translated locally. This confers the possibility of homomers formation. However, forebrain CaMKII consists mainly of heteromers and the holoenzyme can include subunits from the four CaMKII genes and the multiple splice variants of those genes. The presence of different subunits could regulate the localization of the holoenzyme. For instance, subunits containing NLS could confer the possibility of nuclear translocation but, at the same time, would co-assemble with cytosolic subunits that could possibly retard its translocation. The regulation of heteromers composition is an important issue that deserves further investigation in the role of this key protein kinase in memory.

## Author Contributions

GZ and AR proposed the subject, discussed, and wrote the manuscript. NF discussed and revised the manuscript.

## Conflict of Interest Statement

The authors declare that the research was conducted in the absence of any commercial or financial relationships that could be construed as a potential conflict of interest.
